# Exercise and antitumor immunity: molecular signaling, immune dynamics, and remodeling of the tumor microenvironment

**DOI:** 10.3389/fimmu.2026.1888946

**Published:** 2026-08-20

**Authors:** Guanmin Zhang, Qiuju Hu, Haiyang Zou

**Affiliations:** 1 Department of Physical Education, Kangwon National University, Gangneung, Republic of Korea; 2 Department of Physical Education, Henan Technical Institute, Zhengzhou, Henan, China

**Keywords:** antitumour immunity, exercise, metabolic reprogramming, myokines, tumour microenvironment

## Abstract

Exercise is increasingly being investigated as a host-directed physiological intervention capable of modulating cancer-related immune, metabolic, and vascular processes. Epidemiological studies associate higher levels of habitual physical activity with lower cancer risk and improved outcomes in several patient populations, whereas preclinical studies have identified candidate mechanisms involving catecholamines, cytokines, metabolites, extracellular vesicles, immune-cell trafficking, and tumor perfusion. However, the strength of evidence varies substantially across mechanisms and experimental systems. Acute exercise transiently mobilizes natural killer cells and cytotoxic T cells, whereas repeated exercise training may modify systemic inflammation, metabolic homeostasis, and vascular function. Evidence from selected animal models further suggests that these responses can alter immune-cell infiltration, tumor metabolism, and treatment responsiveness. By contrast, direct evidence of sustained remodeling of the human tumor microenvironment remains limited. This review integrates molecular, cellular, and microenvironmental findings while distinguishing established observations from model-dependent mechanisms and proposed cross-tissue pathways. We also examine heterogeneity in exercise regimens, candidate response biomarkers, and priorities for mechanism-informed clinical trials. Standardized exercise reporting, temporally resolved biosampling, and paired systemic and tumor-level analyses are needed to determine when and in which patients exercise produces biologically meaningful antitumor effects.

## 1 Introduction

Cancer is increasingly understood as a systemic disease in which malignant cells interact with immune, metabolic, stromal, and vascular networks within the host (1, 2). Against this background, physical activity and exercise have attracted attention as potentially modifiable host-level exposures. Epidemiological studies associate higher habitual physical activity with a lower incidence of several cancers and with improved outcomes after diagnosis in selected cancer populations (3, 4). In breast cancer survivors, higher postdiagnosis physical activity and increases in activity after diagnosis have been associated with lower all-cause and breast-cancer–specific mortality; however, these observational findings do not establish that exercise directly remodels tumor biology (5, 6). Moreover, a recent review has systematically synthesized the molecular mechanisms and clinical evidence for exercise interventions in breast cancer, providing a useful framework for interpreting these cancer-specific responses (7). Exercise guidelines for cancer survivors primarily support the safety and efficacy of appropriately prescribed aerobic and resistance exercise for improving cancer-related fatigue, physical fitness, physical function, quality of life, and other patient-centered outcomes (3). Thus, clinical recommendations, epidemiological associations, and mechanistic antitumor evidence should be interpreted as related but distinct evidence domains.

Although research in this field has advanced rapidly, a comprehensive understanding of exercise-mediated antitumor mechanisms across biological scales remains lacking. Current evidence points to effects at multiple levels. At the cellular level, acute exercise can rapidly mobilize effector immune cells through the epinephrine–IL-6–natural killer (NK) cell axis (8, 9); at the tissue level, exercise-induced hemodynamic changes may improve tumor perfusion and promote vascular normalization (10, 11); and at the molecular and circulating levels, myokines and extracellular vesicles contribute to interorgan communication between skeletal muscle and distant tissues (12). These findings highlight the antitumor potential of exercise from complementary perspectives. However, the field still lacks a unified framework integrating molecular signaling, immune-cell dynamics, and tumor-microenvironment remodeling. This raises an important question: do exercise-associated antitumor effects arise from the combined action of multiple pathways, or do they reflect broader systemic reprogramming of the host immunometabolic network?

Accordingly, this review has three objectives. First, we summarize evidence for acute exercise responses and repeated exercise-training adaptations using consistent terminology. Second, we evaluate host-to-tumor signaling across molecular, immune, metabolic, stromal, and vascular compartments, explicitly distinguishing direct evidence from mechanistic inference. Third, we identify methodological limitations and propose standardized approaches for exercise prescription, biomarker sampling, and translational study design. Rather than assuming a universal antitumor pathway, we propose that exercise responses depend on exercise dose, tumor type, disease stage, treatment context, and the baseline immune and metabolic state of the host.

## 2 Exercise as a systemic modulator of tumor biology

### 2.1 Terminology and temporal dimensions of exercise responses

The following terminology is used consistently throughout this review. “Acute exercise” refers to a single, planned exercise bout, with outcomes assessed during exercise or within a prespecified post-exercise recovery period (13). For the purposes of this review, acute exercise in preclinical studies denotes a single exercise exposure rather than a repeated training protocol. “Exercise training” refers to repeated, planned exercise sessions delivered over a defined intervention period with the potential to induce physiological adaptation (14). Because intervention durations vary substantially across animal and human studies, we do not apply a universal number of days or weeks to define exercise training. “Habitual physical activity” refers here to free-living physical activity assessed over an extended period and should not be used interchangeably with a controlled exercise intervention. Exercise is considered a planned, structured, and repetitive subset of physical activity (15). The nonspecific term “regular exercise” is avoided unless the exercise mode, intensity, frequency, session duration, intervention length, progression, and adherence are reported (16, 17). **Table 1** outlines terminology and minimum reporting requirements for exercise studies in oncology.

**Table 1:** Terminology and minimum reporting requirements for exercise studies in oncology.

Term	Operational definition	Human-study reporting	Preclinical reporting
Acute exercise	One planned exercise bout, with outcomes assessed during exercise or within a prespecified recovery period (13, 14).	Report mode, intensity, duration, and the exact sampling time during exercise and/or recovery (16, 17).	Report the treadmill or wheel protocol, speed, incline or workload, bout duration, habituation, and the interval between exercise and tissue collection (18).
Exercise training	Repeated planned exercise sessions delivered over a defined intervention period and capable of producing physiological adaptation (14) (16).	Report FITT-VP, progression, adherence, supervision, and timing relative to cancer treatment (3, 16).	Report frequency, total intervention duration, load progression, voluntary versus forced exercise, circadian timing, and timing relative to tumor inoculation and treatment (18).
Habitual physical activity	Free-living bodily movement accumulated over time; it is not equivalent to a controlled exercise intervention (15)	Report the questionnaire or device used, measurement window, intensity categories, sedentary time, and data-processing criteria (19).	Usually not applicable unless spontaneous cage activity or voluntary wheel running is quantified; report monitoring duration and device parameters (18).
Sedentary behavior	Any waking behavior characterized by an energy expenditure of ≤1.5 metabolic equivalents while sitting, reclining, or lying (19).	Report the device or self-report method, wear-time criteria, posture classification, and bout/interruption definitions (19).	Report housing conditions, cage activity monitoring, light/dark cycle, and whether inactivity reflects normal behavior, illness, or protocol-imposed restriction (18).
Prehabilitation	Exercise or multimodal intervention delivered after cancer diagnosis and before definitive or acute treatment to improve functional reserve (20).	Report the interval from diagnosis to treatment, intervention duration, treatment delays, baseline impairments, and perioperative or pretreatment outcomes (20).	Report timing relative to tumor implantation, tumor establishment, surgery, and anticancer treatment; avoid labeling exercise before tumor inoculation as clinical prehabilitation (18).
Concurrent exercise	Exercise delivered during an active course of anticancer therapy (3).	Report synchronization with chemotherapy, radiotherapy, immunotherapy, or endocrine-treatment cycles, including same-day timing and dose modifications (3).	Report the interval between exercise and drug or radiation administration, treatment dose, sampling time, and whether exercise occurred before or after each treatment exposure (18).

Acute exercise produces transient sympathetic, hemodynamic, metabolic, and immune responses (14). Catecholamine release can rapidly mobilize NK cells and differentiated T-cell subsets into the circulation (9). In mouse tumor models, Pedersen and colleagues demonstrated that epinephrine- and exercise-induced IL-6–dependent NK-cell mobilization and redistribution contributed to exercise-associated tumor control (21). In pancreatic ductal adenocarcinoma models, Kurz and colleagues identified an IL-15/IL-15Rα-dependent pathway linking aerobic exercise to intratumoral CD8^+^ T-cell accumulation and function (22). These findings provide direct mechanistic evidence in defined animal models; they do not establish that the same pathways dominate across all cancers or in patients.

**Figure 1:**
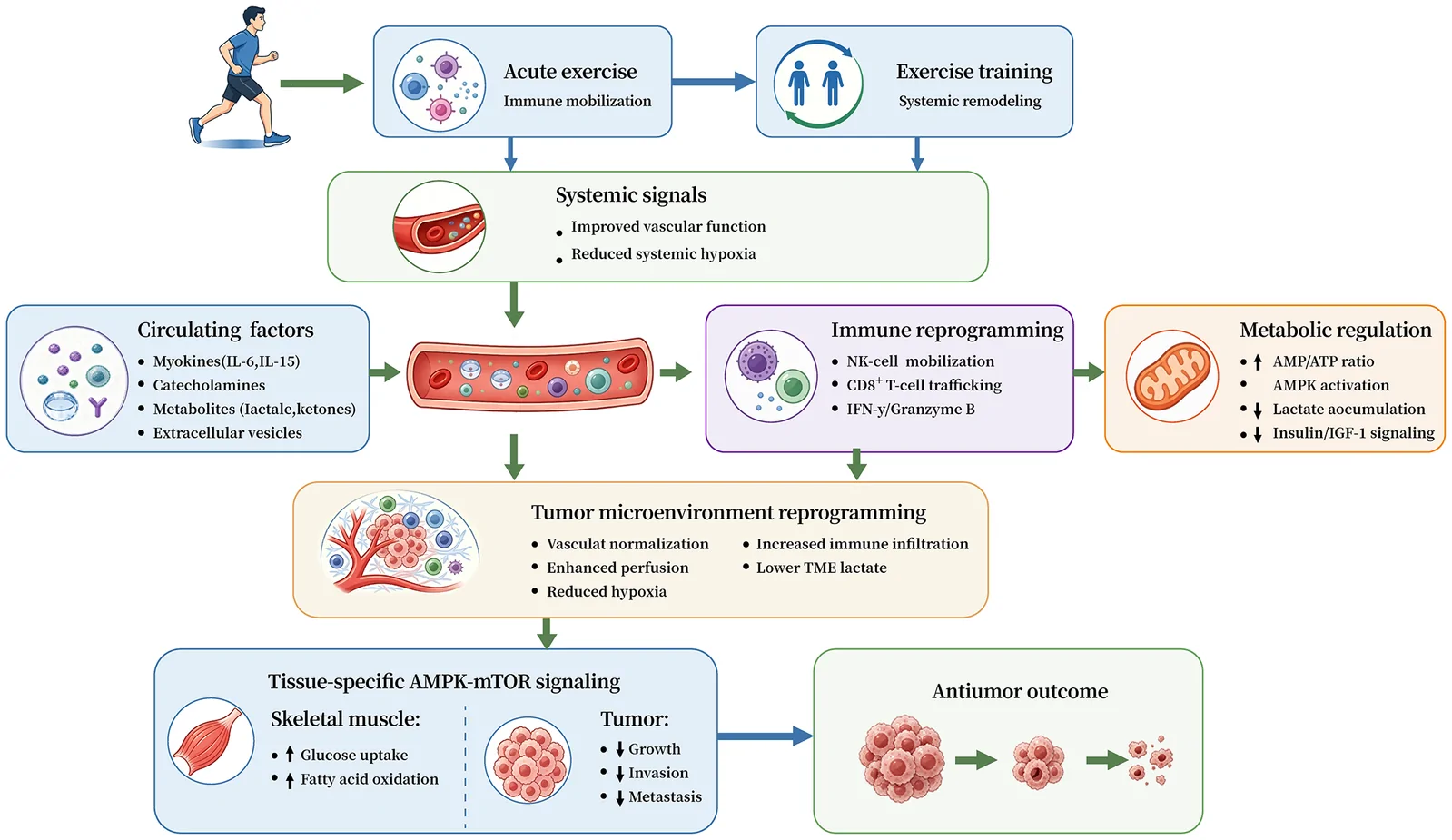
Evidence-based framework for systemic responses to acute exercise and exercise training. Acute exercise produces transient hemodynamic, neuroendocrine, metabolic, and immune responses, whereas repeated exercise training can induce longer-term host adaptations. Solid arrows indicate pathways supported by direct exercise–tumor experiments in defined models; dashed arrows indicate indirect evidence or proposed cross-tissue mechanisms. The relative contribution of each pathway is expected to vary according to exercise dose, tumor type, disease stage, and treatment context.

Repeated exercise training may produce longer-term adaptations in cardiorespiratory fitness, systemic inflammation, insulin sensitivity, skeletal-muscle metabolism, and vascular function (12, 23). Such adaptations could indirectly modify the host environment in which tumors develop or respond to therapy. However, evidence for sustained exercise-induced immune remodeling within human tumors remains limited. Clinical exercise trials in patients with cancer have more consistently demonstrated feasibility and improvements in cardiorespiratory fitness, fatigue, physical function, or quality of life than direct changes in tumor immune composition (24). Acute immune-cell mobilization and training-induced host adaptation should therefore be treated as related but distinct processes. These temporally distinct systemic responses and adaptations are summarized in **Figure 1**.

### 2.2 Endocrine, metabolic, and immune adaptations

Exercise acts as a systemic physiological stimulus that coordinates endocrine, metabolic, and immune adaptations, thereby reshaping the host environment in which tumors develop and respond to treatment. The AMP-activated protein kinase (AMPK)–mechanistic target of rapamycin (mTOR) axis has been proposed as a metabolic checkpoint linking cellular energy availability to immune and tumor-related responses (25). By sensing cellular energy status and coordinating metabolic processes with immune function, this pathway may contribute to exercise-induced systemic adaptation. Exercise also alters circulating hormone profiles and recalibrates host metabolic homeostasis through energy-sensing pathways. Accordingly, the AMPK–mTOR axis may represent an important regulatory link between systemic metabolism and antitumor immunity.

Exercise training can improve peripheral insulin sensitivity and may reduce circulating insulin and components of the IGF axis in selected populations (26). These systemic endocrine changes could decrease growth-promoting signaling in insulin- or IGF-responsive tumors, but direct suppression of tumor PI3K–AKT–mTOR activity should not be assumed without tumor-tissue measurements. In contracting skeletal muscle, increased ATP turnover raises the AMP/ATP or ADP/ATP ratio and activates AMPK (27). AMPK can inhibit mTORC1 through mechanisms that include phosphorylation of TSC2 and subsequent TSC2-dependent inhibition of Rheb (28). These molecular events are well established in skeletal muscle and cell biology; whether the same changes occur in tumor cells after exercise depends on tumor type, exercise protocol, and sampling time.

mTOR signaling regulates lymphocyte activation, differentiation, and metabolic substrate use (29). AMPK contributes to lymphocyte metabolic adaptation during energetic or glucose-limited stress (30). Exercise-associated changes in nutrient availability and systemic metabolism could therefore influence the capacity of effector lymphocytes to function in nutrient-restricted tumors. This possibility should be distinguished from direct evidence that exercise causes tumor-infiltrating lymphocytes to switch to fatty-acid oxidation. In murine melanoma models, aerobic exercise normalized tumor vasculature and increased endothelial VCAM1 expression, whereas its effects on tumor growth, hypoxia, and immune-cell composition differed between YUMMER 1.7 and B16F10 tumors (31). These findings support model-specific vascular and immune remodeling but do not establish a universal AMPK-mediated reduction in metabolic competition between tumor cells and immune cells.

### 2.3 Exercise-induced exerkines and circulating mediators

Exercise alters a broad network of circulating mediators, often collectively termed exerkines. These mediators include cytokines and myokines, catecholamines, bioactive lipids, metabolites, nucleic acids, and extracellular vesicles released from skeletal muscle and other organs (32). Their concentrations and biological effects depend on exercise mode, intensity, duration, training status, nutritional state, and the timing of biospecimen collection (33). Accordingly, no single circulating molecule is likely to explain the full range of exercise-associated tumor responses.

Among the best-studied mediators, catecholamines contribute to rapid immune-cell mobilization (34), while transient muscle-derived IL-6 participates in metabolic and immune signaling (35). IL-15 supports NK- and CD8^+^ T-cell homeostasis (36), and IL-15/IL-15Rα signaling was required for exercise-associated tumor control in murine pancreatic ductal adenocarcinoma models (22). These effects should be distinguished from chronic tumor- or adipose-derived IL-6 signaling, which can have different biological consequences (8).

Other candidate mediators include sphingosine-1-phosphate (S1P), irisin, SPARC, lactate, other metabolites, and exercise-responsive extracellular vesicles. Acute exercise and endurance training can alter circulating S1P (37), although direct evidence connecting exercise-induced S1P changes to tumor control remains limited. Exercise-induced SPARC has been linked to suppression of colon tumorigenesis in an experimental mouse model (38), while irisin has shown direct effects in selected cancer-cell and animal models (39). Exercise also increases the release of extracellular vesicles containing proteins and can modify their regulatory-RNA cargo (40), but the tissue origin of many circulating vesicles and the delivery of specific cargo to tumors remain uncertain. More recently, exercise-induced changes in microbial one-carbon metabolism and circulating formate were shown to enhance CD8^+^ T-cell antitumor activity and immune-checkpoint-inhibitor efficacy in preclinical melanoma models (41). These observations support a network model of host-to-tumor communication, while the relevance of individual mediators in patients remains to be established.

## 3 Integrated molecular mechanisms of exercise-induced tumor suppression

### 3.1 Energy sensing and metabolic signaling

Exercise-induced energetic stress is most directly established in contracting skeletal muscle, where increased ATP turnover and changes in adenine-nucleotide ratios activate AMPK (27, 42). AMPK can inhibit mTORC1 through TSC2-dependent phosphorylation and through direct phosphorylation of raptor, and it can activate PGC-1α-associated transcriptional programs involved in mitochondrial biogenesis, substrate oxidation, and metabolic adaptation (28, 43). These responses are central to skeletal-muscle adaptation but should not automatically be attributed to tumor cells or tumor-infiltrating immune cells.

In selected preclinical tumor models, exercise has been associated with AMPK–raptor–mTOR modulation in hepatocarcinogenesis, redistribution of glucose uptake and oxidation away from breast and melanoma tumors toward skeletal and cardiac muscle, and altered central carbon metabolism in CD8^+^ T cells (25, 44). However, the direction and magnitude of these responses vary according to tumor model and exercise dose; for example, exercise-intensity–dependent tumor control was observed in a murine breast-cancer model, whereas two melanoma models showed different tumor, vascular, and immune responses (45). Moreover, comparison of three exercise protocols in a pancreatic ductal adenocarcinoma model did not identify additional tumor control beyond gemcitabine or significant differences in tumor-infiltrating lymphocytes (46). We therefore propose that systemic metabolic adaptation may influence tumor biology through changes in hormones, nutrients, perfusion, immune-cell function, and circulating mediators, rather than through universal direct activation of tumor-cell AMPK.

The AMPK–mTOR pathway should consequently be presented as a candidate integrative node rather than as a proven initiating mechanism of exercise-induced tumor suppression. Studies that simultaneously measure pathway activation in skeletal muscle, circulating immune cells, and tumor tissue are needed to establish the direction and tissue specificity of this signaling response.

### 3.2 Metabolic reprogramming and mitochondrial redox signaling

Exercise increases reactive-oxygen-species production, particularly in contracting skeletal muscle (47). At moderate and transient levels, ROS can function as signaling molecules rather than solely as mediators of oxidative damage (47). The canonical KEAP1–NRF2 pathway provides one mechanism by which changes in cellular redox state induce antioxidant-response genes. Oxidative or electrophilic modification of redox-sensitive cysteine residues in KEAP1 can inhibit KEAP1-dependent ubiquitination and degradation of NRF2, allowing NRF2 to accumulate, translocate to the nucleus, and induce genes involved in glutathione metabolism, detoxification, and antioxidant defense (48).

The relevance of this pathway to exercise oncology is context dependent. Controlled NRF2 activation can protect normal tissues from oxidative injury, genomic damage, and carcinogenic stress, whereas sustained or constitutive NRF2 activation in established tumors may support antioxidant capacity, metabolic plasticity, tumor progression, and resistance to anticancer treatment (4). Direct demonstrations that exercise activates tumor-cell KEAP1–NRF2 signaling and subsequently suppresses tumor growth are limited. Therefore, exercise-induced redox adaptation should be described as a plausible modifier of tumor and host stress responses rather than a uniformly antitumor mechanism.

Exercise-associated changes in mitochondrial function, oxygen availability, and substrate metabolism may also influence redox balance within the tumor microenvironment. Selected preclinical studies have reported altered tumor glucose use or reduced tumor hypoxia after exercise (25). However, the respective contributions of ROS, NRF2, p53, and MDM2 to these exercise-associated outcomes have rarely been examined within the same experiment. Future studies should use tissue-specific genetic manipulation or pharmacological pathway inhibition to distinguish causal redox mechanisms from downstream associations.

### 3.3 Epigenetic and extracellular-vesicle–mediated regulation

Metabolites such as acetyl-CoA, α-ketoglutarate, NAD^+^, and lactate can influence chromatin-modifying enzymes or provide substrates and cofactors for chromatin modifications, thereby linking cellular metabolism to gene regulation (49). Exercise has been associated with changes in DNA methylation and noncoding RNA profiles in skeletal muscle and circulating samples, whereas evidence for exercise-responsive histone modifications in humans remains comparatively limited (50, 51). However, evidence that these exercise-responsive epigenetic changes occur in tumor cells or directly mediate tumor control remains limited. The tissue and cell type in which an epigenetic change was measured should therefore be stated explicitly.

Exercise also induces a rapid increase in circulating extracellular vesicles and alters their molecular cargo (52). Exercise-related and endurance-associated extracellular-vesicle preparations have contained miRNAs associated with skeletal muscle, including miR-133a and miR-206 (53). Nevertheless, the detection of a muscle-associated miRNA in circulating or urinary vesicle preparations does not by itself establish skeletal-muscle origin, delivery to tumor tissue, or antitumor activity. Statements regarding extracellular-vesicle–mediated muscle-to-tumor signaling should therefore be framed as a developing hypothesis unless tissue-of-origin tracing, recipient-tissue uptake, and functional cargo transfer have been demonstrated.

A more rigorous experimental framework would combine tissue-specific vesicle labeling, temporally resolved plasma sampling, tumor-uptake measurements, and functional blockade of candidate cargo. Recent human work using paired skeletal-muscle, plasma-extracellular-vesicle, and adipose-tissue measurements to investigate exercise-responsive miR-1 illustrates the type of source-to-recipient evidence required, although it does not demonstrate delivery to tumors (54). Until comparable evidence is available in tumor-bearing systems, exercise-induced epigenetic and extracellular-vesicle responses should be interpreted as potential mechanisms of cross-tissue communication rather than established drivers of tumor suppression.

### 3.4 An evidence-based cross-tissue model

**Figure 2:**
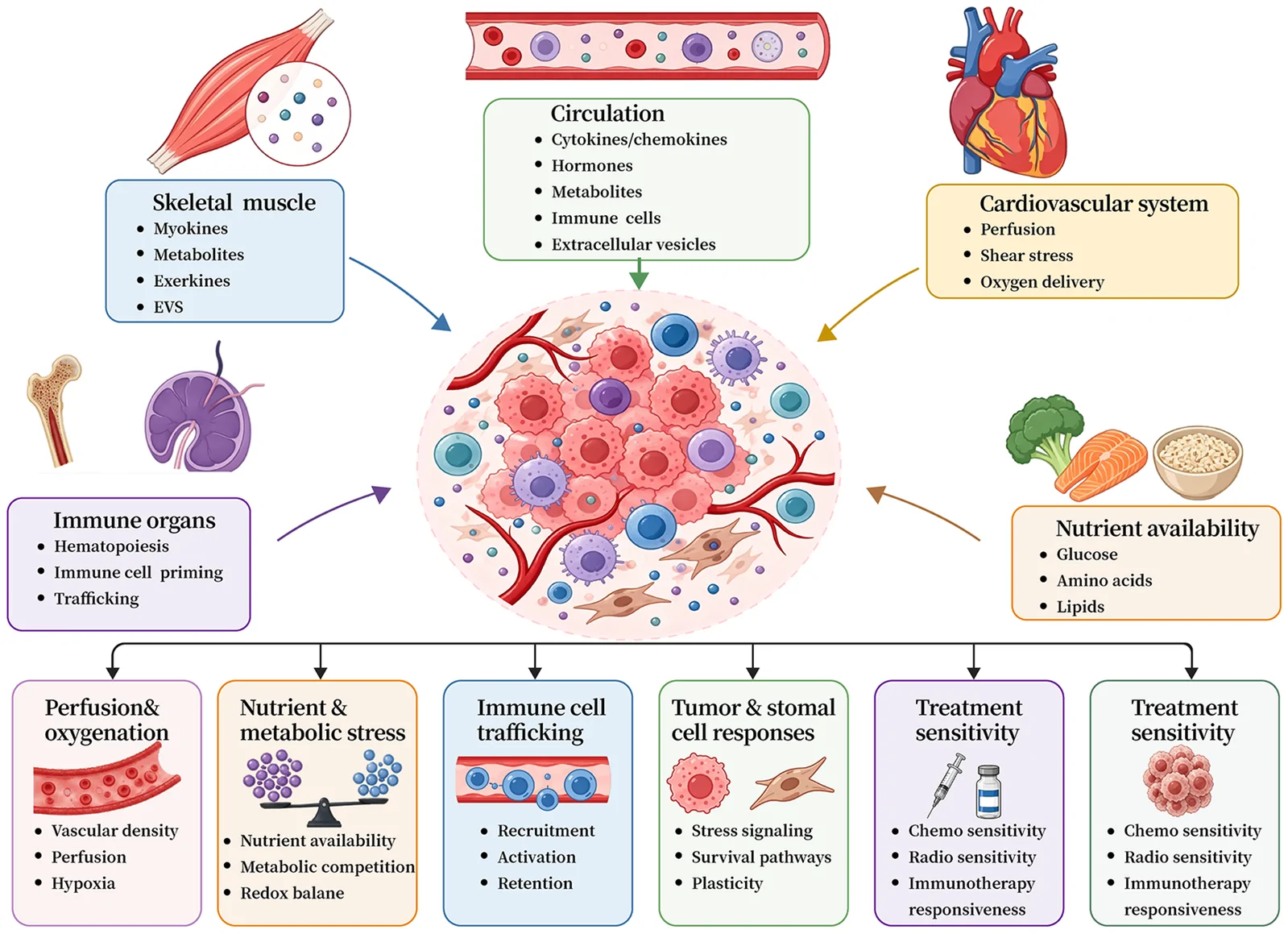
Tissue-specific and proposed cross-tissue mechanisms linking exercise to tumor-related responses. Molecular pathways shown within skeletal muscle are well established components of exercise adaptation. Their effects on circulating immune cells and tumor tissue may be indirect and context dependent. Solid lines denote pathways demonstrated in exercise–tumor experiments; dashed lines denote mechanisms inferred from general molecular biology or non-tumor exercise studies.

On the basis of the evidence summarized above, we propose a cross-tissue model in which exercise-related signals generated in skeletal muscle, the circulation, the cardiovascular system, and immune organs may converge on the tumor microenvironment (55, 56). In this model, changes in perfusion, nutrient availability, cytokines, metabolites, and immune-cell trafficking may alter tumor stress responses and treatment sensitivity (57). The proposed tissue-specific and cross-tissue pathways are summarized in **Figure 2**. This framework does not imply that every pathway is activated simultaneously or that skeletal-muscle signaling is reproduced directly in tumor cells.

The model also predicts substantial heterogeneity. Tumors differ in baseline vascularization, glycolytic dependence, stromal density, antigenicity, and the extent and spatial pattern of immune-cell exclusion (1, 58). Consistent with this prediction, the same aerobic-exercise regimen produced different effects on tumor growth, hypoxia, vascular remodeling, and immune-cell composition in YUMMER1.7 and B16F10 murine melanoma models (31). Consequently, the same exercise stimulus may produce measurable tumor control in one model and little or no effect in another. Direct mechanistic language is therefore reserved for studies that used pathway blockade, cell depletion, genetic manipulation, or other causal interventions. Associations and cross-tissue extrapolations are described as potential or proposed mechanisms.

## 4 Cellular mechanisms underlying exercise-induced tumor suppression

### 4.1 Immune-cell mobilization, trafficking, and function

Acute exercise rapidly mobilizes NK cells and differentiated T-cell subsets into the circulation through sympathetic activation and β2-adrenergic/catecholamine-dependent mechanisms (9). In murine tumor models, blockade of β-adrenergic signaling or depletion of NK cells attenuated exercise-associated tumor control, supporting a causal role for this pathway in that experimental setting (21). However, increased circulating immune-cell numbers do not necessarily indicate increased tumor infiltration or sustained intratumoral function. Human studies should therefore distinguish peripheral mobilization, tumor trafficking, and functional persistence as separate endpoints.

Exercise training has also been linked to CD8^+^ T-cell trafficking in selected preclinical models. In murine breast-cancer models, exercise increased CD8^+^ T-cell infiltration and improved responses to immune-checkpoint blockade through a CXCR3-dependent mechanism (59). This study provides direct support for CXCR3-mediated trafficking in defined breast-cancer models, but the pathway should not be generalized to all tumors. In murine melanoma models, exercise-associated effects on tumor growth, hypoxia, vascular remodeling, and immune-cell composition differed between YUMMER1.7 and B16F10 tumors, indicating that tumor antigenicity and baseline immune context may influence the response (31).

Changes in myeloid-cell states have also been reported, including exercise-associated changes in macrophage MHC-II and inflammatory features in melanoma models and reduced M2-associated macrophage polarization in a high-fat-diet–associated breast-cancer model (60). Because tumor-associated macrophages occupy multidimensional and context-dependent activation states, the terms “M1-like” and “M2-like” are preferable to treating M1 and M2 as fixed, mutually exclusive populations (61). Evidence that exercise consistently promotes dendritic-cell maturation or macrophage reprogramming across tumor types remains insufficient, and negative or neutral findings should therefore be included.

Local metabolic and structural conditions determine whether recruited immune cells retain effector function. Tumor acidity, lactate accumulation, nutrient competition, vascular dysfunction, and dense stroma can limit T-cell trafficking, activation, and cytotoxic function (62, 63). Exercise-associated improvements in tumor perfusion, vascular function, or local metabolic conditions could make selected tumors more permissive to immune-cell entry and function (59, 64). However, direct *in vivo* evidence that exercise reverses established T-cell exhaustion remains limited. The current evidence is therefore stronger for acute immune-cell mobilization than for durable functional reprogramming within human tumors.

### 4.2 Tumor-cell and immunometabolic responses

Exercise may influence tumor cells indirectly through systemic metabolic and endocrine changes and, under *in vitro* conditions, through exercise-conditioned circulating factors. Exercise-induced catecholamines have been shown to activate Hippo-pathway tumor-suppressor signaling and reduce breast-cancer-cell growth in MCF-7 and MDA-MB-231 models (65). Exercise-conditioned human serum has also been evaluated in several defined cancer-cell models. Serum collected after an acute exercise bout reduced the viability of MCF-7 and MDA-MB-231 breast-cancer cells (66), inhibited the growth of LNCaP prostate-cancer cells (67), and reduced the proliferation of LoVo colon-cancer cells in association with IL-6-related regulation of DNA-damage responses (68). These experiments demonstrate that post-exercise blood contains factors capable of altering cultured cancer-cell behavior, but they do not reproduce immune interactions, tissue perfusion, drug metabolism, or the concentrations and exposure durations present within tumors *in vivo*. The exercise protocol, participant population, serum-collection time, serum concentration, exposure duration, and exact cancer-cell model should therefore be reported whenever exercise-conditioned-serum studies are discussed.

Tumor metabolic and cell-intrinsic responses to exercise are heterogeneous. In a murine 4T1 triple-negative breast-cancer model, moderate exercise reduced tumor mass and altered tumor mitochondrial respiratory capacity and the expression of genes related to glycolytic metabolism, although tumor lactate concentration was not reduced (69). In murine melanoma, exercise increased p53-associated pro-apoptotic signaling, ceramide accumulation, and tumor-cell apoptosis in B16F10 tumors but not in BP tumors, demonstrating that tumor-cell responses can differ even between models of the same cancer type (70). By contrast, comparison of high-volume continuous exercise, low-volume continuous exercise, and high-intensity interval training in a murine pancreatic ductal adenocarcinoma model did not demonstrate additional tumor control beyond gemcitabine treatment or significant differences in tumor-infiltrating lymphocytes (46). These contrasting findings indicate that metabolic and tumor-cell responses depend on tumor genotype, model characteristics, exercise dose, intervention timing, and treatment context.

Proposed downstream mechanisms include changes in glucose transport, glycolytic-gene expression, lactate metabolism, HIF-1α signaling, apoptosis, autophagy, and cellular senescence. However, direct evidence currently supports only selected endpoints in defined models, such as altered glycolysis-related gene expression in 4T1 tumors and p53- and ceramide-associated apoptosis in B16F10 melanoma (70). Claims that exercise uniformly suppresses GLUT1 or LDHA, induces AMPK–mTOR-dependent autophagy, or drives tumor-cell senescence are not supported consistently across exercise–tumor studies. These mechanisms should therefore not be presented as one established causal sequence. Each endpoint should be retained only when it is linked to a specific exercise protocol, tumor model, tissue measurement, and mechanistic experiment; otherwise, it should be identified explicitly as a hypothesis or removed.

### 4.3 Stromal, extracellular-matrix, and vascular remodeling

Cancer-associated fibroblasts and extracellular-matrix components can restrict immune-cell migration, elevate interstitial fluid pressure, and contribute to heterogeneous tumor perfusion (71, 72). These stromal barriers are particularly relevant in highly desmoplastic tumors such as pancreatic ductal adenocarcinoma, in which dense extracellular matrix, hyaluronan accumulation, elevated interstitial pressure, and vascular compression can impair tissue perfusion and therapeutic delivery (73). Exercise could potentially influence stromal organization indirectly through changes in tumor perfusion, systemic inflammation, and circulating mediators (57). However, direct evidence that exercise reprograms defined CAF subtypes or consistently reduces tumor collagen deposition, lysyl oxidase activity, or hyaluronan accumulation remains limited.

Evidence for exercise-associated vascular effects is stronger than that for direct CAF reprogramming, although vascular responses remain dependent on tumor type and exercise protocol. In orthotopic 4T1 and E0771 murine breast-cancer models, exercise reduced tumor growth, increased microvessel density, vessel maturity, and tumor perfusion, and decreased intratumoral hypoxia. In the 4T1 model, exercise combined with cyclophosphamide also prolonged tumor-growth delay compared with cyclophosphamide alone (10). Exercise training also normalized tumor vascular features in murine breast-cancer models, promoted CXCR3-dependent CD8^+^ T-cell infiltration, and sensitized immune-checkpoint-blockade–refractory tumors to treatment (59). In YUMMER1.7 and B16F10 murine melanoma models, aerobic exercise normalized tumor vasculature and increased endothelial VCAM1 expression in both models, but its effects on tumor growth, hypoxia, and immune-cell composition differed; increased CD8^+^ T-cell infiltration and tumor-growth suppression were observed in YUMMER1.7 but not B16F10 tumors (31). These findings support vascular adaptation in selected preclinical tumors rather than a universal response across cancer types.

Exercise-induced increases in blood flow and luminal shear stress are established stimuli for systemic vascular adaptation. Repeated shear-stress exposure can enhance endothelial nitric oxide synthase expression or phosphorylation, increase nitric-oxide bioavailability, and improve NO-dependent vasodilation in human and experimental vasculature (23). However, these systemic vascular adaptations should not be assumed to occur identically within tumor endothelium. Exercise-induced upregulation of tumor-endothelial VCAM1 has been demonstrated in YUMMER1.7 and B16F10 melanoma models (31). By contrast, statements that exercise consistently decreases tumor VEGF, increases the Ang-1/Ang-2 ratio, upregulates ICAM-1, or converts immune-excluded tumors into inflamed tumors require direct measurements in the specific tumor model and exercise protocol under discussion. Where such measurements are unavailable, these pathways should be presented as candidate mechanisms rather than established vascular effects.

## 5 Mechanisms of exercise-induced tumor suppression in the microenvironment

### 5.1 Remodeling of the immune microenvironment

**Figure 3:**
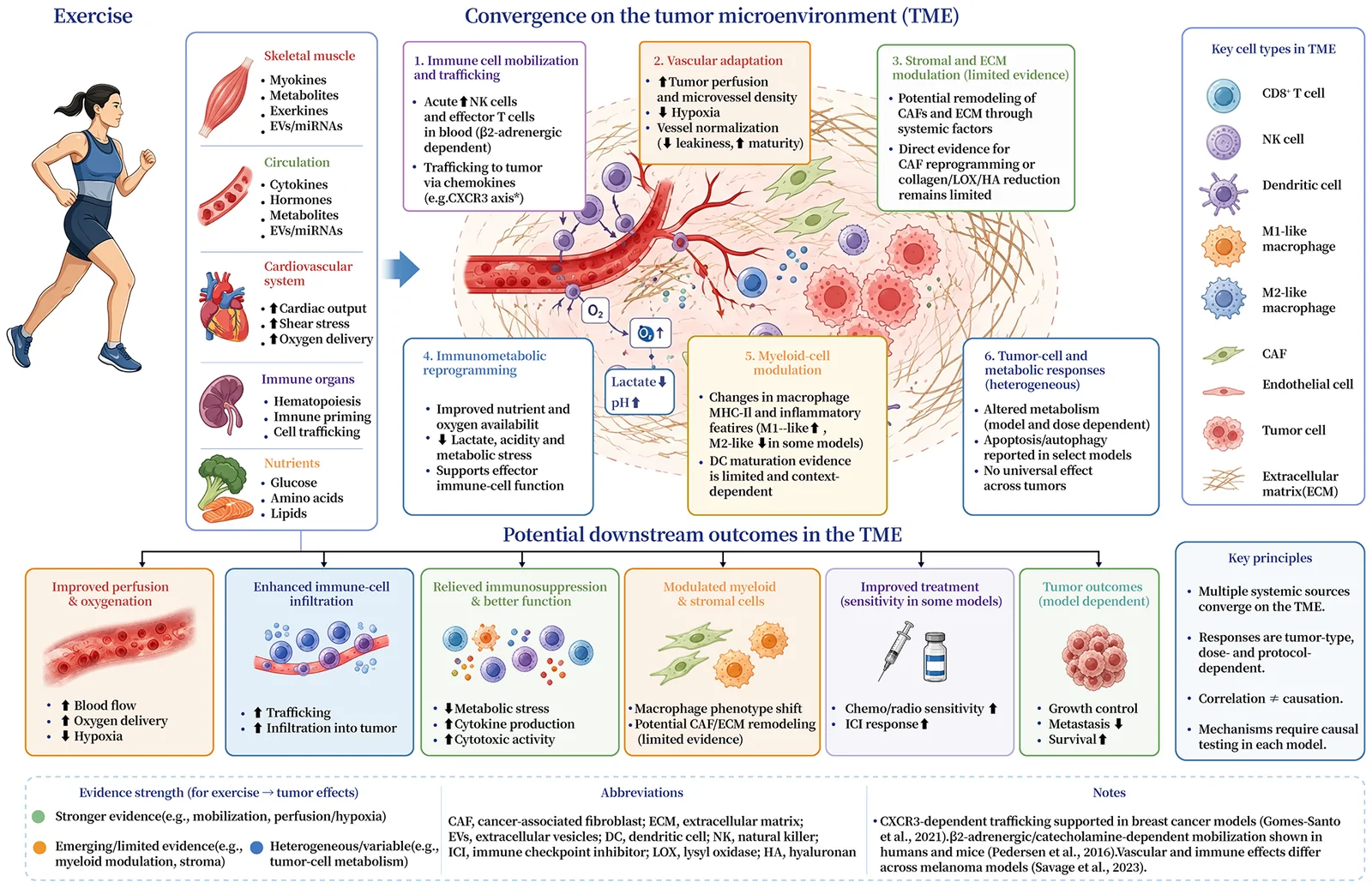
Tumor-ecosystem responses associated with exercise. Evidence is grouped according to immune, metabolic, stromal, extracellular-matrix, and vascular compartments. Solid arrows indicate direct findings from exercise–tumor studies, whereas dashed arrows denote proposed or incompletely validated mechanisms. Responses vary according to tumor type, exercise regimen, and treatment context.

The major tumor-ecosystem responses associated with exercise are summarized in **Figure 3**. Effective antitumor immunity depends not only on the abundance of immune cells but also on their ability to enter tumor tissue and retain effector function within the tumor microenvironment (74). In animal models, exercise mobilizes NK cells through epinephrine- and IL-6-dependent signaling and promotes their redistribution to tumors, thereby contributing to tumor control (21). Exercise has also been associated with increased CD8+ T-cell infiltration and greater expression of cytotoxic mediators such as IFN-γ (75). At the structural level, the CXCL9/10–CXCR3 chemokine axis (76), together with ICAM-1- and VCAM-1-mediated adhesion and transendothelial migration, regulates effector T-cell entry into tumors. Exercise-associated improvements in vascular organization, endothelial function, perfusion, and interstitial pressure may facilitate immune-cell extravasation and infiltration. Collectively, these structural adaptations may render the tumor microenvironment more permissive to immune-cell trafficking (77).

However, immune-cell infiltration does not guarantee sustained antitumor function. Lactate accumulation, acidic pH, nutrient competition, and other local constraints can suppress T-cell activity. Exercise-associated antitumor immunity may therefore involve both increased immune-cell entry and improved maintenance of effector function through changes in the local metabolic environment (78, 79). Although reduced lactate accumulation and acidity could support cytotoxic lymphocyte function, direct evidence that exercise restores established T-cell exhaustion remains limited. The concept of a “niche-permissive effect” captures the possibility that structural and metabolic remodeling makes tumors more receptive to immune infiltration and activity. Exercise-associated formation of tertiary lymphoid structures (TLSs) has been proposed in relation to altered chemokine signaling (80, 81). but this mechanism requires further direct validation. It therefore remains unclear whether exercise primarily enhances immune-cell recruitment, local functional persistence, or both.

### 5.2 Alterations in vascular structure and oxygenation

Abnormal tumor vasculature and heterogeneous perfusion limit immune-cell infiltration and reduce the efficiency of anticancer drug delivery. Vascular disorganization, excessive permeability, and uneven blood flow contribute to tissue hypoxia and elevated interstitial pressure, thereby promoting an immunosuppressive tumor microenvironment. Improving vascular structure and function can reduce interstitial pressure, enhance perfusion, alleviate hypoxia, and facilitate immune-cell entry and the delivery of therapeutic agents (82). In selected preclinical models, exercise has improved tumor perfusion and reduced hypoxia (10), changes that may enhance responses to radiotherapy and chemotherapy (83, 84). Most studies have focused on moderate-intensity aerobic exercise; the vascular effects of other modalities, including resistance and interval training, remain incompletely defined.

Exercise-induced hemodynamic changes increase blood-flow–mediated shear stress and activate endothelial signaling pathways. This response can increase endothelial nitric oxide synthase (eNOS) activity and nitric oxide (NO) production, thereby improving vasodilation and local perfusion.

Reduced hypoxia may promote vascular stabilization by attenuating HIF-1α signaling (85). In some models, this shift may decrease VEGF and selected immunosuppressive signals and alter angiopoietin signaling, including the Ang-1/Ang-2 balance. Such vascular changes could facilitate infiltration by cytotoxic T lymphocytes and NK cells. Reduced CXCL12 expression and increased endothelial ICAM-1 have also been proposed, but these effects should not be generalized without direct measurement in the relevant tumor model. A key question is whether improved vascular function is required for exercise-enhanced immunity across tumor types or is most important in highly hypoxic or poorly perfused tumors (86). Addressing this question will require integrated preclinical and clinical analyses of vascular, immune, and treatment-response endpoints.

### 5.3 Metabolic reprogramming of the tumor microenvironment

Lactate accumulation, tissue acidification, and nutrient depletion in the tumor microenvironment (TME) can constrain antitumor immunity (87). Through aerobic glycolysis, tumor cells consume large amounts of glucose and produce lactate, reducing the availability of metabolic substrates required for effector T-cell activation and cytotoxic function (88). Lactate also acts as a signaling metabolite that can reinforce immunosuppressive conditions (89). Physical activity is closely linked to systemic metabolic health, whereas sedentary behavior is associated with increased cancer risk (90). In selected *in vitro* models, post-exercise serum has inhibited tumor-cell growth and altered metabolic phenotypes (91). Exercise may improve the metabolic landscape of the TME by enhancing perfusion and oxygenation, modifying lactate handling, and redistributing metabolic substrates. These changes could support effector T-cell and NK-cell function and partially alleviate immunosuppressive conditions associated with tumor-associated macrophages and regulatory T cells.

Lactate has context-dependent effects within the tumor microenvironment. Although high lactate concentrations generally suppress effector lymphocyte activity, some immune-cell subsets can use lactate or fatty acids as alternative substrates under specific conditions. Whether exercise-induced changes in circulating or intratumoral lactate promote immunosuppression or metabolic adaptation therefore remains unresolved. AMPK–mTOR and HIF signaling may contribute to exercise-associated metabolic remodeling. AMPK activation can inhibit mTOR signaling and support cellular adaptation to energetic stress, whereas reduced HIF-1α signaling may limit tumor-cell adaptation to hypoxia and glycolytic metabolism. These mechanisms should be interpreted as context dependent rather than universal.

### 5.4 The inflammatory microenvironment and cytokine networks

Tumor-associated inflammation is governed by a dynamic network of cytokines and chemokines, the balance of which shapes the magnitude and quality of local immune responses (22). Exercise can influence systemic inflammatory and immune profiles through myokines and immunoregulatory cytokines, including IL-6, IL-7, and IL-15 (92). IL-6 is particularly context dependent: chronic tumor- or adipose-derived IL-6 signaling is commonly associated with immune suppression and tumor progression, whereas transient pulses of muscle-derived IL-6 during exercise can contribute to anti-inflammatory signaling. These responses include induction of IL-10 and the IL-1 receptor antagonist (IL-1ra) (93). Other candidate exercise-responsive mediators, including irisin and SPARC, have been reported to influence tumor-cell growth or immune-cell activity in selected experimental systems, but their clinical relevance remains uncertain.

Habitual physical activity is associated with lower levels of chronic low-grade inflammation, a recognized contributor to tumor development and progression. Improvements in systemic inflammatory status may, in turn, modify local cytokine and chemokine networks within the tumor microenvironment. Proposed effects include attenuation of TGF-β- and chronic IL-6-centered immunosuppressive signaling and enhancement of IL-15- and IFN-γ-related pathways that support effector lymphocyte function. Exercise may also influence immune-cell trafficking through chemokine networks, including CCL2 and CXCL9/10. However, the direction and magnitude of these changes vary among tumor models. Further studies are needed to determine whether inflammatory network remodeling initiates antitumor immunity or primarily amplifies responses generated through other pathways.

### 5.5 Extracellular matrix remodeling

Excessive deposition and abnormal remodeling of the extracellular matrix (ECM) create major physical barriers to immune-cell infiltration. A dense ECM can form an immune-exclusion barrier (94), restrict T-cell access to the tumor core, and reduce the migration of dendritic cells and effector T cells by increasing tissue stiffness (95). Dense matrix structures can also impede the diffusion of oxygen and therapeutic agents, thereby reinforcing immunosuppression and treatment resistance. Matrix-associated factors, including endostatin, may further influence tumor architecture through matrix–endothelial interactions (96). Because exercise affects vascular function and tissue mechanics, it could improve immune-cell infiltration and drug diffusion by reducing interstitial pressure or tissue stiffness; however, direct evidence for consistent exercise-induced ECM remodeling remains limited.

Mechanistically, exercise-associated changes in tissue mechanics may influence integrin-mediated mechanotransduction, including focal adhesion kinase signaling and downstream transforming growth factor-β pathways. These processes could limit fibroblast activation and attenuate fibrotic matrix formation (97). Exercise may also alter the deposition or organization of ECM components, including collagen I and hyaluronan. However, it remains unclear whether ECM remodeling is a general requirement for exercise-associated antitumor effects or is relevant mainly in highly fibrotic tumors. Future studies should define the timing, magnitude, and tumor-type specificity of these responses using direct matrix measurements and causal interventions.

## 6 Clinical and translational implications

Clinical evidence should be interpreted according to the endpoint assessed. Exercise trials have consistently evaluated feasibility, cardiorespiratory fitness, muscle function, fatigue, quality of life, and treatment tolerance. Fewer studies have incorporated tumor-tissue measurements, and only a limited number have been designed to evaluate recurrence or survival. Improvements in patient-centered outcomes should therefore not be interpreted automatically as evidence of tumor-microenvironment remodeling.

### 6.1 Exercise as an adjuvant therapy to conventional treatment

The role of exercise in cancer treatment is receiving increasing attention. As a potential adjunctive therapy, it can influence the treatment outcome by reshaping the host’s physiological functions and the tumor microenvironment (98). Tumor hypoxia and abnormal vasculature can reduce radiosensitivity (99) and impair drug delivery. Exercise may transiently increase tumor blood flow (100), and, in selected preclinical models, promote vascular normalization and improve perfusion. Increased perivascular cell coverage, reduced interstitial pressure, and improved vessel stability could facilitate the delivery of oxygen and therapeutic agents. Improvements in tumor perfusion and oxygenation have been associated with enhanced responses to radiotherapy and chemotherapy in several experimental models. Nevertheless, vascular and hemodynamic responses to exercise are context dependent, and their clinical significance across tumor types, disease stages, and treatment regimens remains uncertain.

Clinically, the potential benefits of exercise can be considered in two broad domains. First, exercise may complement anticancer therapy by improving tumor oxygenation and drug distribution and by modulating the immune microenvironment, thereby potentially increasing sensitivity to radiotherapy, chemotherapy, and targeted therapy. Second, exercise can improve treatment tolerance and help patients maintain the prescribed treatment dose and schedule. By supporting systemic metabolic health, reducing chronic inflammatory burden, and preserving skeletal-muscle mass and function, exercise can alleviate cancer-related fatigue and may help limit cachexia, thereby improving adherence to treatment (101). Exercise modalities also produce distinct physiological adaptations: aerobic exercise primarily improves cardiorespiratory fitness and oxygen transport, whereas resistance training is particularly important for preserving muscle mass, strength, and metabolic function (24).

Representative clinical trials illustrate the distinction between functional, systemic biomarker, and disease-related endpoints. In breast-cancer survivors, reports from a 16-week combined aerobic and resistance exercise intervention showed attenuation of adipose-tissue inflammation, including changes in adipose-tissue macrophage phenotypes and inflammatory cytokine secretion (102), as well as improvements in metabolic-syndrome measures, sarcopenic-obesity indices, insulin, IGF-1, leptin, and adiponectin (103). These studies evaluated adipose-tissue, metabolic, and body-composition outcomes rather than tumor response or recurrence. In patients with resectable or borderline-resectable pancreatic cancer receiving neoadjuvant therapy, the PancFit trial compared a prescribed home-based aerobic and resistance exercise program with enhanced usual care. Six-minute walk distance improved in both groups, while the prescribed-exercise group completed more weekly strength-training sessions; objectively measured activity, quality of life, and clinical outcomes did not differ significantly between groups (104). In metastatic breast cancer, the multinational PREFERABLE-EFFECT randomized trial demonstrated that a 9-month supervised, structured, and individualized exercise program improved physical fatigue and health-related quality of life, together with selected functional and symptom outcomes (24). By contrast, the phase III CHALLENGE trial showed that a 3-year structured exercise program initiated after adjuvant chemotherapy for stage II or III colon cancer significantly improved disease-free survival, with findings also consistent with improved overall survival (105). These studies differ substantially in cancer type, disease stage, intervention timing, comparator condition, and primary endpoint and should not be treated as equivalent evidence for a single antitumor mechanism.

The effects of exercise may also depend on intervention timing, including prehabilitation before treatment, concurrent exercise during therapy, and rehabilitation after treatment. Future studies should identify the tumor types and treatment settings in which impaired perfusion, metabolic dysfunction, or inflammatory burden constitute modifiable limiting factors and determine whether exercise can causally alleviate these constraints.

### 6.2 Synergistic effects of exercise and immunotherapy

Immunotherapy efficacy is strongly influenced by the tumor immune microenvironment, creating a plausible context in which exercise could exert complementary effects (106). The efficacy of immune-checkpoint inhibitors is often limited by inadequate effector T-cell infiltration, local hypoxia, and metabolic competition. Emerging preclinical evidence suggests that exercise may increase CD8+ T-cell and NK-cell infiltration and alter the spatial immune architecture of tumors (107). By improving perfusion, reducing selected stromal barriers, and modulating chemokine gradients, exercise could help shift some tumors from an immune-excluded toward a more inflamed phenotype (108). Exercise-associated improvements in perfusion and metabolism may also reduce lactate accumulation and glucose competition, creating a more favorable environment for effector T cells (109). However, these mechanisms remain context dependent and are not yet established clinically.

Exercise has a biologically plausible potential to complement immunotherapy by influencing circulating immune-cell availability, tumor perfusion, chemokine signaling, and local metabolism (41, 59). Preclinical studies in breast cancer and melanoma have reported increased CD8^+^ T-cell infiltration or effector activity and enhanced immune-checkpoint efficacy under specific exercise conditions (41). More recent preclinical work has implicated exercise-responsive gut microbial one-carbon metabolism and circulating formate in the promotion of antigen-specific CD8^+^ T-cell function and immune-checkpoint-inhibitor efficacy (41). However, human evidence currently consists mainly of observational associations and feasibility- or supportive-care–focused exercise studies rather than randomized trials powered to determine immune-checkpoint response rates or survival (110). Exercise–immunotherapy synergy should therefore be described as an emerging translational hypothesis rather than an established clinical strategy. Future trials should prespecify exercise timing relative to drug administration, objective exercise-dose and adherence measures, tumor immune endpoints, immune-checkpoint response criteria, and treatment-related adverse events.

### 6.3 Personalized exercise prescription for patients with cancer

Although growing evidence suggests that exercise may improve outcomes in people with cancer (111), the metaphor of “exercise as medicine” is insufficient to guide precision oncology practice. Metabolic and immune responses to exercise vary substantially among patients and are influenced by tumor type, treatment phase, baseline fitness, comorbidities, and treatment-related toxicities. These differences support incorporating exercise into a precision-medicine framework (4). Exercise intensity, frequency, duration, and mode determine not only the conventional training load but also the magnitude and temporal pattern of molecular stimuli (3). For example, the release of myokines, the catecholamine response, and the extent of inflammatory and metabolic regulation may help indicate whether a given exercise dose has crossed a biologically meaningful threshold. However, such thresholds have not yet been validated for tumor-level outcomes.

Existing exercise guidelines for cancer survivors provide an important clinical foundation but should not be interpreted as tumor-specific antitumor prescriptions. Current consensus recommendations support avoidance of inactivity and the use of appropriately individualized aerobic and resistance exercise to improve outcomes such as cancer-related fatigue, physical function, anxiety, depressive symptoms, and health-related quality of life (112). For many common cancer-related outcomes, evidence supports programs involving moderate-intensity aerobic exercise approximately three times per week or combined aerobic and resistance exercise approximately two to three times per week, although the optimal mode and dose vary according to the clinical endpoint, treatment status, baseline fitness, comorbidities, and patient preferences (3). These recommendations are based mainly on evidence concerning safety, symptoms, physical function, fitness, and quality of life; a validated molecular threshold or tumor-response–specific exercise dose has not yet been established (111).

In clinical practice, exercise prescription should be based on multidimensional assessment, including baseline functional capacity, cardiopulmonary status, comorbidities, treatment phase, treatment-related toxicities, and, where available, relevant inflammatory or immune measures. These factors may influence both safety and responsiveness to exercise (113). Exercise should therefore be treated as an adaptive intervention that is adjusted as treatment and clinical status evolve (105). Intensity, volume, and modality may need to be modified in response to symptoms, functional changes, and adverse effects to maintain an appropriate balance between safety, adherence, and physiological stimulus. In this way, exercise can progress from a general lifestyle recommendation toward an adjunctive strategy with quantifiable dosing, dynamic adjustment, and patient stratification.

## 7 Limitations and future directions

### 7.1 Heterogeneity in exercise types and protocols

Exercise studies in oncology use substantially different exposure models. Preclinical studies include voluntary wheel running, forced treadmill or wheel running, swimming, continuous exercise, and interval training (56, 57). These approaches differ in experimental controllability, stress exposure, workload quantification, reproducibility, and behavioral selection (18). Human studies include supervised or home-based aerobic exercise, resistance training, combined aerobic and resistance training, high-intensity interval training, and free-living physical-activity interventions (3). Grouping these distinct protocols under the single label “exercise” obscures potentially important differences in physiological dose, host response, and antitumor mechanism.

Exercise protocols should be reported using the frequency, intensity, time, type, volume, and progression framework (16). Human studies should report how exercise intensity was prescribed and monitored, such as percentage of VO_2_peak, heart-rate reserve, external workload, repetition maximum, or rating of perceived exertion, together with intervention tailoring, progression, supervision, adherence, and permitted co-interventions (17). Preclinical studies should report speed, incline or resistance, bout duration, weekly frequency, intervention length, workload progression, voluntary versus forced exercise, familiarization, circadian timing, handling or aversive-stimulus exposure, and whether exercise began before or after tumor implantation (18). Both clinical and animal studies should specify the timing of individual exercise sessions and the overall intervention relative to chemotherapy, radiotherapy, immunotherapy, surgery, and biospecimen collection (3).

Existing data indicate that the relationship between exercise dose and tumor control cannot be assumed to be linear. In murine breast-cancer models, moderate-intensity exercise delayed tumor growth and reduced tumor burden, whereas lower- and higher-intensity protocols did not produce comparable tumor control; although low- and moderate-intensity exercise both affected vascular features, enhanced CD8^+^ T-cell infiltration and effector activity were observed primarily after moderate-intensity exercise (45). In a murine pancreatic ductal adenocarcinoma model, high-volume continuous exercise, low-volume continuous exercise, and high-intensity interval training did not provide additional tumor control beyond gemcitabine treatment and did not produce significant differences in tumor-infiltrating lymphocytes (46). These findings indicate that null, non-linear, and treatment-dependent responses should be incorporated into exercise-prescription models rather than assuming that greater exercise intensity or volume necessarily produces stronger antitumor effects. **Table 2** outlines Representative exercise protocols and tumor-related outcomes in preclinical and clinical studies.

**Table 2:** Representative exercise protocols and tumor-related outcomes in preclinical and clinical studies.

Preclinical studies														
Study	Model/population		Cancer	Exercise mode		Intensity method	Frequency/duration		Timing relative to cancer/treatment	Mechanistic endpoints			Tumor/clinical outcome	Main limitation
Pedersen et al., 2016 (21)	Tumor-bearing mice across five transplantable or spontaneous models; mechanistic analyses emphasized B16F10 melanoma.		Melanoma and other murine tumors	Voluntary wheel running		Self-selected; wheel distance/activity recorded rather than externally prescribed.	Continuous wheel access; approximately 4 weeks before tumor challenge and maintained during tumor development.		Pre-tumor conditioning plus concurrent exercise during tumor growth.	Epinephrine, exercise-induced IL-6, NK-cell mobilization/redistribution; β-adrenergic blockade, NK depletion, and IL-6 blockade.			>60% reduction in tumor incidence or growth across the tested models; greater intratumoral NK-cell accumulation.	Predominantly prophylactic design; voluntary dose was heterogeneous; mouse findings do not establish the pathway in patients.
Kurz et al., 2022 (22)	Orthotopic and subcutaneous KPC-derived PDAC models and an autochthonous KC model.		Pancreatic ductal adenocarcinoma	Forced treadmill aerobic exercise		15 cm/s for 30 min (mild-to-moderate, protocol-defined workload).	5 sessions/week; short interventions of up to approximately 3 weeks, depending on model.		Initiated shortly after implantation or in established tumors; tested alone and with anti-PD-1 or chemotherapy.	IL-15/IL-15Rα signaling, intratumoral CD8+ T cells, single-cell RNA sequencing, CD8 depletion, IL-15 blockade, and FTY720.			Reduced tumor burden and enhanced CD8+ T-cell–dependent antitumor immunity; improved response to selected therapies.	Short, forced-treadmill murine protocols; workload-related stress and model-specific biology limit clinical extrapolation.
Gomes-Santos et al., 2021 (59)	Multiple murine breast-cancer models, including tumors with limited baseline checkpoint sensitivity.		Breast cancer	Aerobic treadmill training		Moderate-to-vigorous, protocol-defined treadmill workload.	Approximately 30–45 min/session, 5 days/week, for 1–2 weeks.		Exercise and immune-checkpoint therapy were initiated in mice with established tumors.	Tumor vascular normalization, CXCR3-dependent CD8+ T-cell trafficking, intratumoral effector function, and checkpoint blockade.			Improved tumor control and sensitization of otherwise poorly responsive tumors to immune-checkpoint blockade.	Short intervention in murine models; optimal clinical dose and relevance across breast-cancer subtypes remain unknown.
Gomes-Santos et al., 2024 (45)	Tumor-bearing mice with breast cancer; exercise dose compared within the same experimental framework.		Breast cancer	Low-, moderate-, and high-intensity treadmill running		Approximately 30%, 60%, or 90% of individually measured maximal running velocity.	30 min/session for 11 consecutive days after familiarization and maximal testing.		Exercise delivered after tumor establishment; blood and tumor endpoints assessed during and after the intervention.	Vascular features, perfusion, circulating responses, CD8+ T-cell infiltration, CD69, IFN-γ, and granzyme B.			Only moderate intensity delayed tumor growth and reduced tumor burden; low and high intensity were not equivalently effective.	Single short-term murine setting; relative treadmill intensity is not directly interchangeable with human exercise prescription.
Savage et al., 2023 (31)	YUMMER1.7 and B16F10 melanoma-bearing mice.		Melanoma	Aerobic treadmill exercise		Paper-defined moderate fixed workload.	Repeated treadmill sessions over a 2-week intervention.		Exercise delivered during established tumor growth.	Vessel structure/function, endothelial VCAM1, ERK5 S496 phosphorylation, hypoxia, CD8+ T cells, and macrophage features.			Vascular remodeling occurred in both models, but tumor-growth, hypoxia, and immune responses differed; stronger benefit in YUMMER1.7.	Marked model dependence; a common exercise regimen did not produce uniform immune or tumor effects.
Gupta et al., 2022 (46)	Male C57BL/6 mice bearing subcutaneous PDAC4662 tumors, with or without gemcitabine.		Pancreatic ductal adenocarcinoma	Low-volume continuous, high-volume continuous, or high-intensity interval treadmill training		LVCE/HVCE: 12 m/min; HIIT: ten 1-min intervals at 20 m/min alternated with 1 min at 8 m/min.	LVCE: 15 min, 5 days/week; HVCE: 45 min, 5 days/week; HIIT: 3 days/week; 2 weeks.		Started when tumors reached approximately 50 mm³; gemcitabine given 3 days/week.	Tumor growth and tumor-infiltrating lymphocyte composition across three exercise doses.			No additional tumor control beyond gemcitabine and no significant differences in tumor-infiltrating lymphocytes.	Subcutaneous model and short intervention; important negative result but limited power for subtle immune effects.
Phelps et al., 2025 (41)	ICI-resistant BRAF V600E melanoma and B16F10 models; forced-treadmill and voluntary-wheel validation experiments.		Melanoma	Treadmill training with voluntary-wheel validation		Protocol-controlled aerobic workload; intensity reduced after tumor engraftment in prolonged experiments.	One-week acclimation plus approximately 4 weeks of preconditioning, with exercise continued after tumor engraftment; parallel therapeutic protocols.		Prophylactic and post-engraftment exercise; tested with immune-checkpoint inhibition.	Gut microbiota, one-carbon metabolism, circulating formate, NRF2, antigen-specific CD8+ T cells, FMT, antibiotics, and ICI.			Exercise-associated microbial formate enhanced CD8+ T-cell immunity, restrained tumor growth, and improved ICI efficacy.	Complex preclinical causal chain sensitive to diet, microbiota, housing, and protocol; no prospective human validation.
Clinical studies														
Dieli-Conwright et al., 2018 (103)		100 sedentary, overweight or obese breast-cancer survivors randomized to exercise or usual care.	Breast cancer survivorship	Supervised combined aerobic and resistance training	Aerobic exercise at 65–85% of maximum heart rate plus progressive resistance exercise.		3 sessions/week for 16 weeks; reported adherence was 95%.	Post-treatment survivorship; not synchronized with active tumor-directed therapy.			Metabolic-syndrome score, body composition, insulin, IGF-1, leptin, adiponectin, and fitness-related measures.	Improved metabolic syndrome, sarcopenic-obesity indices, and circulating metabolic biomarkers.		No tumor tissue, recurrence, or survival endpoint; selected sedentary overweight/obese survivor population.
Ngo-Huang et al., 2023 (PancFit) (104)		151 patients with resectable or borderline-resectable pancreatic cancer receiving neoadjuvant therapy.	Pancreatic cancer	Pragmatic home-based aerobic and resistance exercise	Individually prescribed moderate-intensity aerobic exercise plus home resistance training.		Target: ≥30 min aerobic exercise on ≥3 days/week and resistance exercise on ≥2 days/week until surgery.	Concurrent with neoadjuvant therapy and before surgical resection.			No tumor-mechanistic primary endpoint; 6-min walk distance, activity, strength sessions, quality of life, and feasibility.	Walking capacity improved in both groups; intervention increased strength-session participation but showed limited between-group clinical differences.		Enhanced-usual-care comparator, home-program adherence variability, and insufficient power for tumor or survival outcomes.
Hiensch et al., 2024 (PREFERABLE-EFFECT) (24)		357 patients with metastatic breast cancer in a multinational randomized controlled trial.	Metastatic breast cancer	Supervised, structured, individualized aerobic, resistance, and balance exercise	Individualized according to fitness, symptoms, treatment, and metastatic sites.		Nine-month intervention; generally 1-hour supervised sessions twice weekly during the initial 6 months, with continued support.	Delivered during treatment for metastatic disease.			Patient-reported fatigue and quality of life; physical function, cardiorespiratory fitness, strength, pain, and dyspnea.	Improved physical fatigue and health-related quality of life, with benefits in selected functional and symptom outcomes.		Open-label supportive-care trial; tumor response and survival were not primary endpoints.
Courneya et al., 2025 (CHALLENGE) (105)		889 patients with resected stage II or III colon cancer after adjuvant chemotherapy.	Colon cancer	Behaviorally supported, predominantly aerobic exercise program	Target increase of approximately 10 MET-h/week above baseline (roughly 150 min/week of moderate activity).		Three-year intervention with frequent coaching early and tapered contact thereafter.	Initiated after completion of adjuvant chemotherapy, generally within 6 months.			Physical activity and fitness; no paired tumor-tissue mechanistic assessment.	Significantly longer disease-free survival (reported hazard ratio 0.72), with findings consistent with improved overall survival.		Selected post-chemotherapy survivors; behavioral intervention without tumor sampling limits mechanistic attribution and generalizability.

CD8+, cluster of differentiation 8-positive; FMT, fecal microbiota transplantation; ICI, immune-checkpoint inhibition; IFN-γ, interferon gamma; IGF-1, insulin-like growth factor 1; LVCE, low-volume continuous exercise; HVCE, high-volume continuous exercise; HIIT, high-intensity interval training; MET-h, metabolic-equivalent hours; NK, natural killer; NRF2, nuclear factor erythroid 2-related factor 2; PDAC, pancreatic ductal adenocarcinoma; VCAM1, vascular cell adhesion molecule 1. Protocol details are condensed from the primary reports. Cross-study comparisons should account for differences in tumor model, exercise-dose definition, intervention timing, comparator condition, and primary endpoint.

### 7.2 Biomarkers for monitoring exercise responses

No biomarker has yet been prospectively validated to determine whether a patient has received an adequate antitumor exercise dose or is likely to derive a tumor-level clinical benefit. Under standard oncology methodology, classification as a predictive biomarker requires prospective evidence that biomarker status identifies differential benefit from an intervention rather than merely correlating with prognosis or biological respons (114). Candidate measures can nevertheless be organized into distinct categories. Exercise-exposure markers include heart rate, external workload, oxygen consumption, rating of perceived exertion, accelerometry, and intervention adherence; these measures should be reported together with exercise frequency, intensity, time, type, volume, and progression (17). Systemic-response markers include catecholamines, IL-6, IL-15, selected exerkines, lactate and other metabolites, insulin and IGF-related signaling, and extracellular-vesicle profiles (22, 33). Immune-response markers include circulating NK-cell abundance and cytotoxicity, differentiated CD8^+^ T-cell subsets, CXCR3-dependent trafficking, IFN-γ, granzyme B, and defined myeloid-cell phenotypes (45, 59). Tumor-level candidate markers include immune-cell density and spatial localization, perfusion, hypoxia, vascular density and maturity, tumor lactate or metabolic activity, stromal organization, and pathway activation within defined endothelial, immune, stromal, or malignant-cell populations (31, 59).

These candidate measures serve different purposes. Some verify that exercise was delivered at the prescribed physiological dose, some indicate an acute or training-induced systemic biological response, and others may function as pharmacodynamic markers of tumor-level change. They should not be described as predictive biomarkers unless they have been shown prospectively to identify patients who derive differential clinical benefit from an exercise intervention (114). Peripheral blood measurements should not be assumed to represent immune, vascular, metabolic, or transcriptional changes within tumor tissue, supporting the use of paired systemic and intratumoral assessments when feasible (3). Many metabolites, cytokines, immune-cell populations, and extracellular-vesicle components change rapidly during exercise and recovery; sampling time relative to the final exercise bout is therefore a major source of biological and analytical variation (33, 52).

Future trials should use standardized sampling schedules that distinguish the chronic training-adaptation state from the acute response to the most recent exercise bout. Depending on the study question, relevant time points may include a rested baseline, immediate post-exercise sampling, early and delayed recovery, and a post-training assessment obtained after a standardized interval without exercise (33). Paired blood and tumor measurements should be prioritized in window-of-opportunity studies because they permit direct comparison of systemic exposure with tumor-cell, stromal, vascular, and immune changes (3). Imaging methods such as dynamic contrast-enhanced MRI, dynamic hypoxia-sensitive PET, and other validated perfusion assessments can provide complementary spatial and longitudinal information, but acquisition, analysis, and repeatability must be standardized (115). Candidate biomarkers should be evaluated for analytical validity, short- and long-term within-person variability, exercise-dose responsiveness, association with paired tumor-level changes, and prospective prediction of clinically meaningful outcomes (114, 115). **Table 3**. outlines candidate biomarkers and measurement strategies for exercise–oncology studies.

**Table 3:** Candidate biomarkers and measurement strategies for exercise–oncology studies.

Exercise-exposure and systemic-response candidates							
Candidate category	Representative measures	Intended role	Biospecimen/assessment		Recommended timing	Interpretation and main limitation	Supporting evidence
Exercise exposure	Heart rate; external workload; VO_2_ or VO_2_peak; heart-rate reserve; rating of perceived exertion; accelerometry; adherence; progression.	Confirms delivery of the prescribed physiological and behavioral exercise dose.	Wearable monitor; ergometer/treadmill output; metabolic cart; accelerometer; session and adherence logs.		Continuous or per-session recording; summarize across the intervention.	Exposure/fidelity measure only. It does not demonstrate systemic or tumor response.	Garber et al., 2011 (16); Slade et al., 2016 (17)
Systemic endocrine and cytokine response	Catecholamines; IL-6; IL-15/IL-15Rα; selected exerkines.	Characterizes acute systemic signaling linked to immune mobilization and tissue crosstalk.	Plasma or serum assays; selected receptor or cellular measurements where justified.		Rested baseline; immediate post-exercise; early and delayed recovery; standardized post-training rest interval.	Highly time-sensitive. Acute blood changes should not be assumed to represent sustained tumor exposure.	Pedersen et al., 2016 (21); Kurz et al., 2022 (22); Contrepois et al., 2020 (33)
Systemic metabolic response	Lactate; glucose; insulin; IGF-related signaling; lipids; amino acids; targeted or broad metabolomic profiles.	Measures acute substrate use and chronic metabolic adaptation.	Plasma or serum biochemistry, targeted assays, or metabolomics; standardize fasting and recent diet.		Fasted/rested baseline for training adaptation; acute and recovery samples for exercise-bout responses.	Systemic change does not establish intratumoral metabolic change. Treatment and diet are major confounders.	Contrepois et al., 2020 (33); Dieli-Conwright et al., 2018 (103)
Extracellular-vesicle and circulating RNA response	Extracellular-vesicle concentration, size, protein cargo, and miRNA profiles; circulating exercise-responsive miRNAs.	Candidate measure of tissue crosstalk and acute or chronic molecular response.	Standardized plasma preanalytics; vesicle isolation/characterization; RNA or protein profiling.		Baseline; immediate post-exercise; multiple recovery points; standardized post-training sampling.	Preanalytics strongly affect results. A muscle-associated miRNA does not prove muscle origin or tumor delivery.	Whitham et al., 2018 (52); Contrepois et al., 2020 (33)
Circulating NK-cell response	NK-cell abundance; differentiated subsets; activation; degranulation; cytotoxicity.	Measures catecholamine-sensitive mobilization and peripheral functional capacity.	Flow cytometry; absolute counts; cytotoxicity or degranulation assays with standardized target cells.		Pre-exercise; immediate post-exercise; recovery; rested post-training assessment.	Peripheral mobilization is not equivalent to tumor infiltration or persistent intratumoral function.	Graff et al., 2018 (34); Pedersen et al., 2016 (21)
Immune and tumor-level candidates							
Circulating CD8^+^ T-cell and trafficking response	Differentiated CD8^+^ T-cell subsets; CXCR3; activation markers; IFN-γ; granzyme B.	Assesses acute mobilization and candidate trafficking or effector programs.	Flow cytometry; intracellular cytokine and functional assays; receptor-expression analysis.	Acute pre/post/recovery sampling plus rested post-training sampling; pair with tumor tissue when possible.		CXCR3 and effector signals are model-dependent and should not be generalized across cancers.	Graff et al., 2018 (34); Gomes-Santos et al., 2021 (59); Gomes-Santos et al., 2024 (45)
Myeloid-cell phenotype	Monocyte subsets; macrophage MHC-II and inflammatory features; other defined myeloid states.	Explores changes in innate immune composition and activation state.	Multiparameter flow cytometry; immunohistochemistry; transcriptomics; single-cell profiling.	Rested baseline and post-training; add acute sampling only when mobilization is the primary question.		Avoid fixed M1/M2 classification. Phenotypes require tissue-, cell-, and model-specific definition.	Graff et al., 2018 (34); Savage et al., 2023 (31)
Tumor immune density and spatial localization	Intratumoral NK cells; CD8^+^ T-cell density; spatial localization; CXCR3-related trafficking; cell-specific IFN-γ and granzyme B.	Candidate pharmacodynamic evidence that exercise altered immune access to or function within the tumor.	Paired biopsy/resection tissue; multiplex immunohistochemistry; spatial profiling; flow cytometry; single-cell analysis.	Window-of-opportunity baseline and post-intervention tissue; standardize biopsy timing after the last exercise bout.		More informative than peripheral counts, but density alone does not prove durable function or clinical benefit.	Pedersen et al., 2016 (21); Kurz et al., 2022 (22); Gomes-Santos et al., 2021 (59); Savage et al., 2023 (31)
Tumor perfusion, hypoxia, and vascular phenotype	Perfusion; blood flow; hypoxia; microvessel density; vessel maturity; endothelial adhesion molecules; vascular imaging.	Candidate pharmacodynamic assessment of vascular adaptation and spatial heterogeneity.	Tumor histology/molecular assays; dynamic contrast-enhanced MRI; dynamic PET; validated perfusion or hypoxia imaging.	Standardized baseline and post-intervention assessment; establish repeatability before use as an endpoint.		Responses vary by tumor model and protocol. Imaging acquisition and analysis must be prespecified.	Betof et al., 2015 (10); Gomes-Santos et al., 2021 (59); Woodall et al., 2021 (115)
Tumor molecular, metabolic, and stromal response	Tumor lactate or metabolic activity; pathway activation in defined cells; gene-expression changes; stromal organization.	Tests whether systemic exercise exposure is associated with a measurable tumor-level biological response.	Paired tumor tissue; spatial or single-cell profiling; targeted metabolite assays; histology; prespecified pathway analysis.	Pre- and post-intervention tissue paired with blood/imaging; record interval since the last exercise session.		Exploratory unless analytically validated and linked prospectively to clinical outcome. Blood cannot substitute for tumor measurement.	Ligibel et al., 2019 (116); Betof et al., 2015 (10); Savage et al., 2023 (31)

None of the listed measures has been prospectively validated as a predictive biomarker of tumor-level benefit from exercise. Exposure measures verify intervention delivery; systemic and immune measures characterize biological response; tumor-tissue and imaging measures are candidate pharmacodynamic endpoints. Sampling should distinguish acute exercise responses from chronic training adaptation and standardize fasting status, treatment timing, and the interval since the final exercise bout.

### 7.3 Integration of multi-omics and systems biology

Exercise affects the host and tumor through a cross-tissue, multilevel regulatory network. Skeletal muscle, the immune system, the circulation, the gut microbiome, and the tumor microenvironment communicate through exercise-responsive cells and soluble mediators (117). Multi-omics integration is therefore essential for resolving this network (118). For example, combining skeletal-muscle transcriptomics, circulating metabolomics, immune phenotyping, and tumor profiling may help trace how exercise-induced signals propagate from peripheral tissues to the tumor microenvironment. Interpretation must distinguish transient responses to a single exercise bout from adaptations to repeated training, because these processes differ in timing and biological significance. Single-omics analyses or isolated pathway studies are often insufficient to explain the heterogeneous responses observed among individuals and tumor types exposed to similar exercise stimuli (119, 120).

Multi-omics integration also provides a framework for identifying exercise “responders” and “nonresponders.” Because multigene and multi-omics associations are frequently correlational, network-based analyses and causal-inference methods should be combined with experimental perturbation to identify regulatory drivers. The tumor microenvironment is spatially heterogeneous (121), future studies should therefore use spatial omics to characterize exercise-associated immune-cell recruitment and metabolic remodeling with greater anatomical resolution (122). High-dimensional data and limited sample sizes create additional analytical challenges. Robust study design, external validation, interpretable computational methods, and careful control of batch effects will be required to improve the reproducibility and translational value of multi-omics research in exercise oncology.

Multi-omics studies should be organized around biological compartments and prespecified sampling times rather than treating measurements from blood, skeletal muscle, immune cells, the gut microbiome, and tumor tissue as interchangeable. A useful framework would combine skeletal-muscle transcriptomics or proteomics, plasma metabolomics and exerkine profiling, circulating immune-cell phenotyping, stool metagenomics or metabolomics, tumor-tissue spatial or cell-resolved profiling, and longitudinal clinical-outcome assessment (12, 33). Acute post-exercise responses should be analyzed separately from resting post-training changes measured after a standardized interval without exercise. Human time-series profiling has shown that metabolites, cytokines, proteins, and other circulating signals change rapidly and with distinct recovery kinetics after a single exercise bout, whereas longitudinal multi-tissue studies identify adaptations that develop over weeks of training (12). Without this temporal separation, transient exercise-bout or recovery responses may be misclassified as stable training adaptations.

The exercise–microbiota–formate–CD8^+^ T-cell pathway provides an example of how multi-compartment analysis can identify and experimentally test a candidate causal chain. In murine melanoma models, exercise altered microbial one-carbon metabolism, increased circulating formate, enhanced antigen-specific CD8^+^ T-cell activity, restricted tumor growth, and improved immune-checkpoint-inhibitor efficacy (41). Similar multi-omics studies should incorporate pathway-interruption and rescue experiments—such as microbiota depletion or transfer, metabolite supplementation, receptor or pathway blockade, and immune-cell depletion—because correlations across multiple molecular layers do not establish directionality or causality. The relevance of this pathway to human tumors remains to be tested prospectively (41).

## 8 Conclusions

Exercise elicits coordinated neuroendocrine, immune, metabolic, and vascular responses. The strongest mechanistic evidence supports rapid immune-cell mobilization after acute exercise and model-specific changes in immune trafficking, perfusion, and metabolism in selected preclinical tumors. Exercise training also provides established benefits for fitness, fatigue, physical function, and quality of life in patients with cancer, while emerging clinical evidence suggests that disease-related outcomes may improve in selected settings. Important mechanistic uncertainties remain. Skeletal-muscle activation of AMPK–mTOR or redox pathways cannot be assumed to occur in tumor cells, and evidence for consistent exercise-induced CAF reprogramming, extracellular-matrix reduction, tertiary lymphoid-structure formation, or restoration of exhausted T cells in human tumors is currently insufficient. Exercise effects are likely to depend on tumor type, exercise dose, intervention timing, treatment background, and host metabolic and immune status. Future research should use standardized exercise terminology and FITT-VP reporting, include negative and neutral studies, and distinguish acute responses from training adaptations. Mechanistic trials should combine objective exercise-dose measurement with standardized biosampling, paired blood and tumor assessments, spatial or single-cell analyses, and clinically relevant endpoints. These approaches are needed to determine whether candidate pathways are causal, identify patients most likely to respond, and translate exercise from a general supportive recommendation into a mechanism-informed component of cancer care.
